# Editorial: Changing backgrounds and groundbreaking changes: Gynecological surgery in the third decade of the 21st century

**DOI:** 10.3389/fsurg.2022.1060503

**Published:** 2022-11-02

**Authors:** Rafał Watrowski, Stoyan Kostov, Radmila Sparić

**Affiliations:** ^1^ Department of Obstetrics and Gynecology, Helios Hospital Müllheim, Müllheim, Germany; ^2^ Faculty Associate, Medical Center–University of Freiburg, Freiburg, Germany; ^3^ Department of Gynecology, University Hospital “Saint Anna”, Medical University of Varna, Varna, Bulgaria; ^4^ Clinic for Gynecology and Obstetrics, University Clinical Centre of Serbia, Belgrade, Serbia; ^5^ Faculty of Medicine, University of Belgrade, Belgrade, Serbia

**Keywords:** gynecological surgery, surgical complication, pregnancy, laparoscopy, placenta increta, primum non nocere, lymphadenectomy, contained morcellation

**Editorial on the Research Topic** Changing backgrounds and groundbreaking changes: Gynecological surgery in the third decade of the 21st century By Watrowski R, Kostov S and Sparić R. (2022) Front. Surg. 9: 1060503. doi: 10.3389/fsurg.2022.1060503

In the third decade of the 21st century, gynecological surgeons are faced with new technical developments but also with new expectations. The surgical evolution is reflected by individualized approaches to patients and treatments (1), increasing role of “omics” and advanced imaging methods for surgical decisions (2, 3), the substantial shift from vaginal to laparoscopic concepts in urogynecology (4–6), increasing role of the robotic-assisted surgery or increased awareness about surgical complications and their prevention (7).

These favorable developments do not eliminate unresolved problems of the past (e.g., the role of the human factor in surgical complications) and add new pitfalls and uncertainties, e.g., the controversy about laparoscopic tissue morcellation (7), the doubts about the oncological risks and long-term results of minimally invasive procedures following the LACC trial (8–10), or issues regarding fertility preservation in oncological patients (11).

We welcome several high-quality articles within this Research Topic that perfectly illustrate these chances and challenges. The publication by *Alkatout* et al*.* introduces this topic by showing the milestones and pitfalls of gynecological laparoscopy from its beginnings to the present day. This contribution opens our Research Topic and can serve as a compendium of historical knowledge, but also as a source of reflection that technical evolution alone does not absolve its pioneers from personal defeats and that each stage of development of gynecology brings with it challenges and risks that were unpredictable in the early days of laparoscopy. *Alkatout* et al*.* remember in the first sentence of their article that the maxim ‘primum non nocere’, originating from the Hippocratic tradition, remains relevant also with regard to current and future developments in surgery. Notably, we live in a time when many of the principles of the Hippocratic Oath—that formed a bedrock of medical ethics for centuries—are being increasingly ignored or overshadowed by non-scientific and non-medical concepts. Gratifyingly, and in keeping with the Research Topic, many authors have focused on the modern implementation of Hippocarates' principle: ‘When dealing with illness, practice two things: either help or do not harm the patient’ (12).

The papers published in this Research Topic address different laparoscopical approaches (classical, natural orifice, single-port), different areas of gynecologic surgery (urogynaecology, oncology) or deal with specific problems that are the price for the development of minimally invasive approaches, e.g., morcellation risks or safety concerns when performing laparoscopy in pregnancy. Importantly, all authors have proposed constructive solutions that go beyond simply reporting of difficulties. For instance, *Tinelli* et al*.* describe the management of an external iliac vein injury during laparoscopic pelvic lymphadenectomy, supplementing a didactic surgical video with case report and literature review; *Wang* et al*.* evaluated the utility of a novel multi-port system for contained laparoscopic morcellation; *Yang* et al*.* developed a nomogram predicting lymph node metastasis in early-stage cervical cancer.

One of the hallmarks of modern gynecological surgery is the changed paradigm of the surgical approach to pelvic organ prolapse (POP). While vaginal techniques, including transvaginal mesh applications, flourished in the late 20th century, laparoscopic concepts have rapidly evolved over the past two decades. Modern surgeons “discovered” fixation points for mesh or autografted tissue at different levels of the lateral pelvic wall, leading to the development of techniques like pectopexy or laparoscopic lateral suspension (LLS), both applicable *via* the classical and robotic laparoscopic approaches, and thus significantly expanding the therapeutic spectrum for POP beyond the laparoscopic sacrocolpopexy. *Dällenbach* compares the LLS vs. laparoscopic sacrocolpopexy for apical pelvic organ prolapse, and postulates the LLS as the new “gold standard”. We are confident that this not entirely uncontroversial opinion can stimulate scientific debate and sharpen the senses of clinicians regarding the best current treatment for POP (4, 6).

A very special feature of this Research Topic is that every second article is dedicated to surgical interventions in women who are pregnant or want to preserve their fertility. The latter aspect in regard to young patients diagnosed with cervical cancer has been reviewed by *Theofanakis* et al. The challenge of any surgical procedure during pregnancy is that the preservation of the pregnancy should not significantly compromise the safety of the mother or the effectiveness of the treatment (13). In line with these considerations is the preliminary study of *Han* et al*.* reporting of positive experiences with laparoscopic removal of adnexal mass during pregnancy. The study by *Zhang* et al*.* applies a retrograde perspective to analyze peripartum outcomes in 12 patients with a prior transluminal endoscopic transvaginal natural opening (vNOTES), including 10 cases of vaginal delivery and 2 cases of cesarean section. A vaginal fornix incision during vNOTES does not appear to compromise the safety and feasibility of vaginal delivery in a subsequent full-term pregnancy. We believe this small study will enhance interest in vNOTES and stimulate larger, multi-center evaluations. Finally, two papers are dedicated to two serious complications of pregnancy and childbirth (which often occur concomitantly): the spectrum of placenta increta and placenta previa. *Fu* et al*.* describes a parallel loop binding compression suture as an an effective and safe method to reduce postpartum bleeding in women with placenta previa complicated with placenta increta. *Guan* et al*.* presents the results of a multi-center study evaluating the innovative treatment of placenta increta left *in situ* by high-intensity focused ultrasound ablation. Both articles show therapeutic options of potentially vital importance.

We thank all authors who contributed to this research topic—those whose contributions were accepted, but also those whose contributions were rejected—for their efforts and their openness to reviewer comments. We thank the reviewers for their insightful comments and constructive criticism. Finally, we thank the editorial team for their support in handling the manuscripts.

We sincerely hope that the works that constitutes this Research Topic will help clinicians make the right decisions, inspire researchers to further evaluate their surgical practice, and in view of the work of *Alkatout* et al*.* encourage someone to re-read the original Hippocratic Oath.
